# Improvement of depressive symptoms, after a suicide attempt, with dextromethorphan/bupropion combination treatment in a patient with treatment‐resistant depression and psychiatric comorbidities

**DOI:** 10.1002/ccr3.7045

**Published:** 2023-03-07

**Authors:** Bruno Pedraz‐Petrozzi, Michael Deuschle, Maria Gilles

**Affiliations:** ^1^ Department of Psychiatry and Psychotherapy, RG Stress, Central Institute of Mental Health, Medical Faculty Mannheim University of Heidelberg Mannheim Germany

**Keywords:** bupropion, comorbidity, depression, dextromethorphan, suicidal ideation

## Abstract

We admitted a 42‐year‐old patient with severe treatment‐resistant depression and with psychiatric comorbidities. The patient attempted suicide 5 weeks after admission. Subsequently, we initiated dextromethorphan/bupropion based on prior evidence. As a result, the patient demonstrated an improvement in mood symptoms and a reduction in suicide risk, leading to her discharge.

## 1 BACKGROUND

According to recent data from the World Health Organization (WHO), depression is one of the most frequent mental health conditions, with around 300 million people worldwide suffering from it in 2017. This represents a significant financial burden for health systems, with increased disability‐adjusted life years.^1^ About one‐third of people with depression exhibit psychiatric comorbidities, such as panic disorders, phobias, generalized anxiety disorders, posttraumatic stress disorder (PTSD), and personality disorders,^2^, ^3^ representing a challenge for psychiatric treatment. Different worldwide mental health societies and well‐known guidelines suggest that the first‐line intervention for depression consists of antidepressant treatment (e.g., serotonin reuptake inhibitors) combined with cognitive‐behavioral psychotherapy (CBT) in severe cases. However, about 30% of people with depression show a lower to no response,^4^ and thus there is a need for pharmacological alternatives or strategies, including off‐label treatments, for treatment‐resistant depression (TRD) to achieve clinical remission in non‐responders, such as treatment with intravenous ketamine. A recent example of the development of novel strategies against TRD is the pharmacological combination of dextromethorphan (DXM) and bupropion.

DXM is a nonopioid antitussive agent metabolized rapidly by CYP2D6 in the liver, achieving low bioavailability and therapeutic plasma levels after administration.^5^, ^6^, ^7^ Similar to ketamine, DXM is an uncompetitive and non‐selective N‐methyl‐D‐aspartate (NMDA) receptor antagonist^8^ and acts additionally upon the serotonin and norepinephrine transporters, as well as sigma‐1‐receptors.^6^ When combined with bupropion, a dopaminergic‐noradrenergic antidepressant and a CYP2D6 inhibitor, DXM concentrations significantly increase due to pharmacokinetic inhibition in the liver, thus prolonging its pharmacologic effects in the central nervous system (CNS).^6^ The combination DXM/bupropion has been described to rapidly ameliorate depressive symptoms due to NMDA antagonism.^9^

On August 18th, 2022, the Food and Drug Administration (FDA) in the United States of America (USA) approved this combination strategy for treating major depressive disorders.^8^, ^10^, ^11^ However, in Germany, this combination strategy is currently an off‐label treatment, and its use for the treatment of depression is underreported. Additionally, the clinical effects of DXM/bupropion in people with depression and high suicidal risk or psychiatric comorbidities remain unknown. To this end, we present a case of a 42‐year‐old female patient with recurrent depressive disorder and psychiatric comorbidities (PTSD and borderline personality disorder [BPD]) who attempted suicide 5 weeks after admission. The patient was subsequently treated with DXM/bupropion, which resulted in a clinical improvement in mood symptoms and suicide risk, and was discharged afterward.

## 2 CASE PRESENTATION

We report the case of a 42‐year‐old Caucasian female patient diagnosed with recurrent severe depression without psychotic symptoms [ICD‐10: F33.2]. The patient also had PTSD (PTSD Checklist for DSM‐V: 39 points) and BPD (International Personality Disorder Examination: fear of abandonment, unstable relationships, inner emptiness, and intense anger). The patient was hospitalized due to an exacerbation of depressive symptoms, including depressed mood, loss of energy, anhedonia, insomnia, fatigue, hopelessness, and suicidal thoughts.

In 2015, the patient was first diagnosed with a severe depressive episode. Before the first depression diagnosis, the patent completed school and worked as an office clerk. After the first depressive episode, the patient has been diagnosed with psychiatric comorbidities, BPD, and PTSD. In 2018, the patient stopped working due to depression and received a disability pension from the state. In total, the patient has been treated seven times at two different inpatient units, including ours. Concerning PTSD and BPD, the patient has been receiving psychotherapeutic treatment in an outpatient psychotherapeutic service parallel to psychiatric outpatient treatment. There is no known relevant mental illness in the patient's family.

Between 2015 and 2022, the patient underwent several psychopharmacological treatments (citalopram, escitalopram, mirtazapine, sertraline, lithium, venlafaxine, duloxetine, promethazine, quetiapine, pregabalin, modafinil, and doxazosin) that showed low to no clinical effect. Additionally, before the current inpatient treatment, intravenous ketamine was administrated 10 times, but it did not have a sufficient therapeutic effect and was therefore discontinued.

Upon admission, a mental state examination was performed, which revealed that the patient was oriented to place, situation, and time, but had mild limitations in cognitive functions, including memory, attention, concentration, and comprehension. The patient's gait and upper extremity movements were normal, but their speech was slow with brooding content, and their affect was generally restricted. Although no signs of delusions or obsessions were observed, the patient described their current mood as “permanent depressive,” with feelings of hopelessness and irritability. The patient denied any behaviors or willingness to attempt actions against their life but reported having permanent suicidal thoughts, which were not concrete (e.g., “it would be good if I could fall asleep forever and never wake up”). The patient presented intact insight into their illness, adequate judgment, and showed motivation for therapy. Physical, laboratory (including complete blood count and biochemical tests), electrocardiographic, electroencephalographic, and magnetic resonance imaging (MRI) examinations showed no clinical abnormalities.

Initially, the patient was initially treated with bupropion 450 mg/d and trazodone 200 mg/d showing partial effectiveness. For that reason, repetitive transcranial magnetic stimulation therapy was carried out. However, this did not lead to further clinical improvement.

**FIGURE 1:**
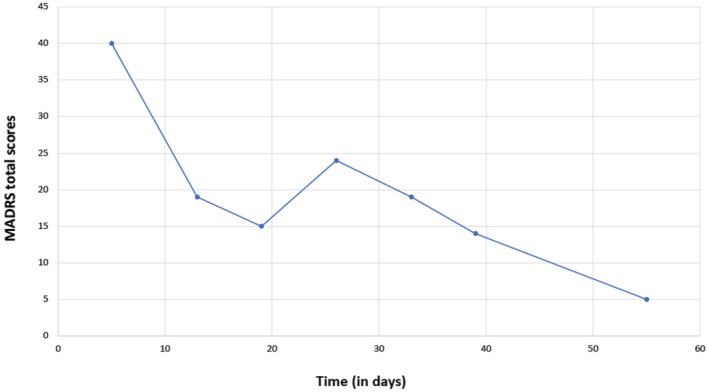
Montgomery–Åsberg Depression Rating Scale (MADRS) total scores during the inpatient treatment at our affective disorders unit. Of note is the date of the suicide attempt (day 0), the beginning of the DXM/bupropion treatment (day 6), and the rapid symptom reduction under this combination treatment. At the week of discharge from the affective disorders unit, she presented a MADRS of 5 points (day 55).

Five weeks after admission, the patient attempted suicide by taking a medication overdose. After intensive medical monitoring in a general hospital, the patient was returned to our unit, showing an exacerbation of depression symptoms (Montgomery–Åsberg Depression Rating Scale or MADRS: 40 points, Figure 1). Columbia Suicide Severity Rating Scale (C‐SSRS) was carried out, obtaining a high risk for suicide (suicide behavior: categories 1–5 answered with “yes”, intensity of suicide behavior: 22).

Upon readmission, the MADRS was conducted, finding values corresponding to a severe depressive episode (Figure 1). Blood biochemical tests, blood count tests, and ECG evaluations were negative. Previous treatment with bupropion was continued, and trazodone was tapered out due to lack of effectiveness. Instead, DXM was given in combination with bupropion. The decision was based on the pharmacokinetic properties of the combination strategy and the previous clinical trials with DXM/bupropion mentioned above. Furthermore, according to our previous experience with the patient in our affective disorders unit, other combinations with bupropion in the past (e.g., bupropion + modafinil, bupropion + quetiapine) did not show clinical effectiveness.

Therefore, we initiated treatment with 30 mg DXM and 450 mg bupropion, subsequently reducing bupropion to 150 mg/d as serum levels had become toxic. No other pharmacological therapies were added during this combination strategy.

A week and a half after beginning DXM/bupropion, the patient showed more energy, established better contact with others, and participated more in ward activities. After 2 weeks, the patient began to sleep without interruption and requested removal of *pro‐re‐nata* sleep medication. Three weeks after beginning the combination treatment, we observed more energy, motivation, an improvement in affective resonance, and a reduction in depressive mood and suicidal thoughts (the patient continuously marked question 9 of the Beck Depression Inventory‐II, or BDI‐II, with a score of 0). Having demonstrated an improvement in depressive symptoms and suicidal thoughts, the patient successfully passed a weekend tolerance test at home without suicidal attempts or thoughts. Five weeks after beginning the combination treatment, we observed a marked improvement in anhedonia, loss of interest, daily fatigue, and concentration, which had been an issue even before the suicide attempt with trazodone. At this point, a second C‐SSRS was carried out, which found a low risk of suicide (suicide behavior: categories 1–5 answered with “no”; intensity of suicide behavior: 2; absent suicide behavior; medical damage for attempt: 0; potential lethality: 0). A transient increase in blood pressure (140/90 mmHg) was observed during the first 2–3 weeks of treatment, but it was self‐limited. Apart from that, no adverse effects were observed during the DXM/bupropion combination therapy. Following clinical improvement of the depression and suicidal thoughts, as well as a satisfactory tolerance test during weekends at home, we discharged the patient for ambulatory follow‐up treatment. Additionally, after the improvement, the patient decided to start occupational therapy at the local community's Occupational therapy workshop. Laboratory tests and ECGs conducted upon discharge showed no clinical abnormalities, and there were no signs of acute suicidality or suicidal thoughts.

## 3 DISCUSSION

To the best of our knowledge, there are no case reports or studies regarding DXM/bupropion treatment and its potential to reduce depressive symptoms and suicide risk in patients with comorbid depression, PTSD, and BPD. In 2016, Lauterbach et al. reported on the first patient with severe depression and comorbidity (generalized anxiety disorder), who was successfully treated with DXM/bupropion (resulting in a 26‐point reduction of BDI‐II scores—from 32 to 6 points—within 21 days).^12^ In our case, under DXM/bupropion treatment, the patient exhibited a 26‐point reduction in MADRS scores within 6 weeks and a reduction in C‐SRSS suicide risk. Therefore, DXM/bupropion treatment appears to be an effective treatment for suicidal thoughts and depressed mood. After clinical improvement, the patient was able to establish a daily routine and develop a future perspective by choosing to attend ambulatory occupational therapy.

Following several clinical trials (e.g., ASCEND and GEMINI trials), the FDA approved the combination of DXM/bupropion for the treatment of major depressive disorder.^8^, ^10^, ^11^ In a clinical trial against placebo (GEMINI trial), the combination of DXM/bupropion demonstrated superiority over the placebo group by significantly reducing MADRS scores as early as week one and achieving symptom remission in almost 40% of cases.^9^ Similar to the GEMINI trial, two other clinical trials with bupropion and DXM/bupropion demonstrated that the combination of DXM/bupropion was superior to bupropion alone^13^, ^14^, ^15^ by showing a MADRS reduction of up to week 1–2 of treatment and a remission rate of 60.5% at week 6 in one clinical trial^15^ and a 47% clinical remission at week 6 in the other (ASCEND trial).^13^, ^14^ However, all clinical trials with DXM/bupropion excluded patients with a significant risk of suicide. Therefore, studies on the effect of DXM/bupropion on suicidal thoughts or behaviors were lacking.^9^, ^14^, ^15^

During clinical trials with DXM/bupropion, dizziness, nausea, dry mouth, decreased appetite, and anxiety were reported as common side effects, with a mild to moderate intensity.^15^ In our patient, we observed a temporary increase in blood pressure, which reached values of 140/90 mmHg and reduced within 2–3 weeks. Apart from that, no adverse effects were observed during the treatment of DXM/bupropion, and no psychiatric complications (e.g., suicide attempts) were observed in our patient, consistent with the adverse effect profile of the reported clinical trials.^9^

The present case report demonstrates the positive effect of the combination therapy DXM/bupropion in reducing severe depressive symptoms after stressful events, such as suicide attempts. Furthermore, it highlights the benefit of NMDA antagonists in combination with antidepressant treatment (bupropion) as an adequate strategy for treating TRD. However, this case report has limitations that must be considered. First, the study design is a case report without random patient selection, so it cannot establish causality. Currently, the DXM/bupropion combination strategy is only approved in the US (Auvelity®) and is distributed as a fixed‐dose combination of 105 mg bupropion and 45 mg DXM. In Europe and Germany, this combination treatment is not yet approved and is considered off‐label. Consequently, the fixed‐dose combination is not currently available, and the two substances were administered separately but simultaneously. Finally, we could not measure the DXM serum concentration, preventing a conclusion on the pharmacokinetic properties of the DXM/bupropion combination strategy.

To summarize, DXM/bupropion combination therapy appears to effectively treat depressive symptoms and suicidal behavior in patients with depression and comorbid psychiatric conditions, such as PTSD or BPD. However, more studies are necessary to demonstrate the efficacy of symptom reduction in these patients.

## 4 TAKE‐AWAY MESSAGE

DXM is an antitussive agent and an NMDA antagonist that, when combined with bupropion, exhibits antidepressant properties^9^ and can achieve remission within 6‐7 weeks.^8^, ^10^, ^11^ While there are few reports regarding the improvement of patients with depression and psychiatric comorbidities, this case demonstrates the effectiveness of DXM/bupropion in reducing depressive symptoms and suicidal risk in a patient with depression and comorbidities (PTSD and BPD).

## AUTHOR CONTRIBUTIONS

Bruno Pedraz‐Petrozzi wrote the introduction, performed “Figure 1”, summarized and wrote the case presentation section, wrote the discussion section, and worked on all other parts of the manuscript preparation, including proofreading. Michael Deuschle supervised the case evaluation, contributed to mentoring and conceptualization, and worked on proofreading the manuscript. Maria Gilles supervised the case evaluation, contributed to the organization of the case report, mentoring, and conceptualization. She also participated in the preparation, writing and proofreading the manuscript.

## FUNDING INFORMATION

The following case report was not supported financially by any pharmaceutical enterprise or industry. The authors of this case report did not receive funding from any public or private organization for this work.

## CONFLICT OF INTEREST STATEMENT

The authors declare no conflicts of interest.

## CONSENT

Written informed consent was obtained from the patient to publish this report in accordance with the journal's patient consent policy.

## Data Availability

The data sets generated and analyzed during the study are not publicly accessible due to the applicable data protection law of the State of Baden‐Württemberg.
